# The Influence of Elevated Oxygen Input and LED Lighting on the Bioactive Compounds Profile of *Lactuca sativa* L. cultivars ‘Carmesi’ and ‘Lugano’

**DOI:** 10.3390/foods15010015

**Published:** 2025-12-20

**Authors:** Augustina Sandina Tronac, Mihaela Dragoi Cudalbeanu, Simona Marcu Spinu, Zina Paraschiv, Sorin Marius Avramescu, Alina Ortan, Sorin Mihai Cimpeanu

**Affiliations:** 1 Faculty of Land Reclamation and Environmental Engineering, University of Agronomic Sciences and Veterinary Medicine of Bucharest, 59 Marasti Boulevard, 011464 Bucharest, Romania; augustina.tronac@yahoo.com (A.S.T.); mihaela.dragoi@fifim.ro (M.D.C.); simona.spinu@fifim.ro (S.M.S.); alina.ortan@fifim.ro (A.O.); mscimpeanu@yahoo.fr (S.M.C.); 2 Faculty of Biotechnologies, University of Agronomic Sciences and Veterinary Medicine of Bucharest, 59 Marasti Boulevard, 011464 Bucharest, Romania; zina.paraschiv22@bth.usamv.ro; 3 Faculty of Animal Productions Engineering and Management, University of Agronomic Sciences and Veterinary Medicine of Bucharest, 59 Marasti Boulevard, 011464 Bucharest, Romania

**Keywords:** lettuce extract, polyphenolic compounds, UHPLC, DPPH, FRAP, CUPRAC, antioxidant index

## Abstract

Enhancing bioactive compounds with antioxidant activities has always been a pursuit of growers in the hydroponic production of lettuce in greenhouses with artificial lighting. Light-Emitting Diode (LED) lighting represents an effective lighting strategy that promotes the accumulation of bioactive compounds in lettuce by reducing dark periods under unfavorable meteorological conditions and extending the photosynthetic duration. In this study, the accumulation of bioactive compounds and their antioxidant activities, using lettuce-adapted growth technologies such as elevated oxygen concentration and elevated oxygen combined with LED red-blue light, were investigated. These technologies were compared to the control, which consisted of the natural oxygen concentration, in a controlled hydroponic system, for the two lettuce cultivars, Carmesi and Lugano. Our results demonstrate that lettuce grown under elevated oxygen combined with LED lighting exhibited increases of up to 48% in total phenolic content and up to 87% in total flavonoid content, depending on cultivar and growing season, compared to control growth technology. The highest antioxidant capacity was recorded under the EOC+LED growth technology, as confirmed by DPPH, FRAP, and CUPRAC assays. This study proposes an effective growth strategy for hydroponic lettuce cultivation that enhances bioactive compound accumulation and provides theoretical and technical guidance for energy-efficient greenhouse production.

## 1. Introduction

Lettuce (*Lactuca sativa* L.) is regarded as one of the cultivated vegetables worldwide [1]. *L. sativa* L. belongs to the *Asteraceae* family, alongside other edible plants such as dandelions, chicory, and tarragon. Originating from the Mediterranean region and now cultivated worldwide, lettuce grows and develops in an annual cycle, either in open spaces or greenhouses, under various conditions. Lettuce contains up to 95% water and various bioactive compounds, including polyphenols, minerals, fiber, and vitamins [2]. Therefore, it can be used not only as a food source but also as a medicinal herb. Furthermore, innovative technologies have been developed to improve the overall content and bioactivities of biochemical compounds [3]. In 2023 alone, the EU produced 3.14 million tons of lettuce and chicory, with Romania accounting for 1.880 tons [4].

Hydroponics is a soilless system of plant cultivation that uses a nutrient-rich liquid solution to submerge the roots, making it the most suitable system for greenhouse conditions. Alternatively, the hydroponics systems have become increasingly widespread, especially since the introduction of LED lighting for plant growth [5]. Additionally, innovative greenhouses equipped with artificial lighting can significantly increase lettuce biomass and quality by precisely controlling environmental factors, such as light, temperature, moisture, humidity, carbon dioxide, and nutrients [6,7]. LED lighting offers numerous advantages over traditional lighting, making it an ideal solution for indoor lettuce growth [8]. Specific wavelengths of LED lighting, in special red-blue light, combined with higher intensity, energy efficiency, a longer lifespan, and reduced heat output, enhance lettuce growth. Adjusting LED light parameters can influence lettuce quality and increase levels of bioactive compounds with biological activities, such as antioxidant activity [9,10]. Alongside LED lighting research, recent studies on elevated oxygen levels, rhizosphere metabolism, and antioxidative mechanisms indicate that increasing dissolved oxygen in the root zone, such as through nanobubbles or microbubbles, can improve leaf nutritional quality, delay senescence, and enhance growth performance in lettuce grown in hydroponic systems [11]. Furthermore, oxygen-enhanced amendments, such as oxygen nanobubble nutrient solutions or oxygen-enriched biochars, can reduce root hypoxia and affect root physiological functions, providing a mechanistic explanation for the observed agronomic improvements [12,13,14]. Lastly, research on antioxidative regulation in lettuce suggests that oxidative signals, including controlled hydrogen peroxide application, stimulate phenolic compound accumulation and increase antioxidant activity [15]. A lack of antioxidant capacity has been associated with quality issues under specific conditions, such as heartburn [16].

Polyphenols are a class of bioactive compounds with strong antioxidant activity that are mainly produced by the plant kingdom. Generally, plants produce them as a defensive mechanism against pathogens, predators, and ultraviolet radiation. Otherwise, these compounds exhibit diverse biological activities, including antimicrobial, antidiabetic, anti-inflammatory, and antiallergic activities [17]. Lettuce contains a wide range of bioactive compounds, including phenolic acids, flavonoids, carotenoids, and vitamin-based antioxidants, which enhance nutritional quality and play key roles in protecting plant tissues from oxidative stress [18]. Understanding how these compounds are regulated is important because their levels can vary significantly in response to environmental factors such as light intensity and spectrum, temperature shifts, and oxygen levels in the root zone [19,20]. Previous studies have shown that high irradiance or spectral imbalances can increase oxidative stress and the production of phenolic and antioxidant compounds [21,22], whereas restricted oxygen supply or poor aeration in hydroponic systems can alter redox balances and influence the accumulation of bioactive compounds [23]. Based on these known relationships, phenolic and antioxidant compounds serve as indicators of plant stress response and nutritional quality, enabling us to evaluate how environmental conditions affect lettuce’s bioactive compounds. Eating lettuce regularly confers numerous health benefits due to its bioactive compounds, including flavonoids and phenolic acids [24].

Nitrate by itself is relatively non-toxic, and small amounts can have a beneficial health impact. When consumed, however, it can be endogenously converted into nitrite. Nitrite is a toxic substance that can react with amines and amides to form N-nitroso compounds, which can lead to methemoglobinemia and an increased risk of cancer [25]. For this reason, nitrate residue in food is regulated in the European Union by Commission Regulation (EC) No 1881/2006. For example, lettuce grown in greenhouses and harvested between 1 October and 31 March should have a nitrate content of less than 5000 mg nitrate/kg fresh weight. Studies indicate that the nitrate levels in hydroponic lettuce can reach a range considered dangerous to human health. Nitrate levels in lettuce leaves are crucial for food safety. LED light can reduce the accumulation of nitrate levels in lettuce compared to natural sunlight [26].

The agricultural sector, due to the decline in arable land, has begun using innovative technologies for growing lettuce under various conditions. Building on the knowledge gaps outlined in the introduction, this study investigates how various growing systems influence the biosynthesis of bioactive compounds in two lettuce cultivars and their potential impact on biological activities such as antioxidant activity. It also proposes a new lighting strategy that combines higher oxygen levels with LED lighting in greenhouses to regulate the accumulation of key phytochemicals in lettuce. The research focuses on three control conditions: lettuce grown under natural oxygen levels, elevated oxygen concentrations, and elevated oxygen combined with LED lighting. By comparing these scenarios, it assesses how environmental factors influence biological activities like antioxidant capacity. Ultimately, producing health-promoting bioactive compounds offers new insights into optimizing sustainable, resource-efficient cultivation practices to enhance lettuce nutritional quality.

## 2. Materials and Methods

### 2.1. Plant Material and Cultivation Conditions

The two lettuce (*Lactuca sativa* L.) cultivars ‘Carmesi’ and ‘Lugano’ were grown in the Research Center of Quality Control of Horticultural Products, University of Agronomic Sciences and Veterinary Medicine, Bucharest (coordinates: 44.4710° N, 26.0656° E). The lettuce was grown using a nutrient film technique (NFT), a type of hydroponic system. Two study years were conducted, in the winters of 2023 and 2024. The certified seeds were sown, and after 8 days, the seedlings were transferred into a Jiffy growing block made of 24 mm diameter peat pellets (Jiffy Products of America Inc., Lorain, OH, USA). The blocks were made with steam-sterilized peat, fertilizers, and pH regulators. The lettuce growth cycle was 40 days.

For precise environmental monitoring, the lettuce cultivation area is equipped with high-precision sensors, including the Aranet CO_2_ sensor (TDSPSPC005), Aranet temperature and humidity sensors (TDSPT509), and a global radiation solarimeter (Aranet PAR sensor, TDSPAR02—Aranet, Hopkins, MN, USA). To ensure proper oxygenation, a SERA AIR 550 R PLUS pump (Sera, Heinsberg, Germany) was employed, and dissolved oxygen levels were continuously monitored using dissolved oxygen sensors (DOS). Moreover, the pump had a low energy consumption of approximately 8 W, which provided an airflow rate of 9.2 L min^−1^ and 552 L h^−1^. Additionally, the nutrient solution was maintained at a pH of 6.3, with an electrical conductivity (EC) adjusted to 2.2 dS/m.

### 2.2. Experimental Design

A 100 W LED lighting system (Kathay Waterproof Plant Grow LED 100 W—Kathay Technology Industrial, Hong Kong, China) with an IP67 water resistance, an input voltage of 220 V, and an operating frequency of 50/60 Hz was used to maintain illumination throughout the growth cycle period. Two LED light bands were used for the illumination process: red, with a peak wavelength of 660 nm, and blue, with a peak wavelength of 450 nm. The ratio of red to blue was 50:50. Lettuce was exposed to light for 14 h per day. The photosynthetic photon flux density (PPFD) values for lettuce cultivars during the two cultivation cycles were as follows: for the Lugano cultivar, the PPFD was 448.66 µmol m^−2^ s^−1^ in the first cycle and 460.65 µmol m^−2^ s^−1^ in the second cycle. In contrast, for the Carmesi cultivar, PPFD was 438.66 µmol m^−2^ s^−1^ in the first cycle and 457.10 µmol m^−2^ s^−1^ in the second cycle.

Three types of lettuce growth technologies were used. The first type served as a control, with lettuce grown under natural oxygen levels (NOC). The second type was lettuce grown under elevated oxygen concentrations (EOC), and the third type was lettuce grown under elevated oxygen concentrations combined with LED light (EOC+LED). All growth technologies were conducted under identical ambient greenhouse light conditions. Supplemental red–blue LED lighting was applied exclusively to the EOC+LED growth technology. Light intensity was continuously monitored using the PAR sensor.

Under NOC conditions, lettuce was grown under ambient oxygen levels corresponding to natural oxygen saturation in the hydroponic nutrient solution. The EOC and EOC+LED conditions were achieved by continuous aeration with an air pump, thereby maintaining higher oxygen availability in the root zone throughout the cultivation period. Oxygen levels were regularly monitored to maintain stable conditions, and any minor fluctuations remained within the hydroponic system’s normal operational range.

### 2.3. Extraction of Lettuce Bioactive Compounds

The lettuce materials underwent a series of procedures, including lyophilization and grinding. The lyophilization procedure was selected due to its generally higher retention of antioxidant compounds compared to simple air-drying [17].

The extraction of bioactive compounds from lettuce materials was performed using the Milestone ETHOS Easy equipment (Milestone SRL, Bergamo, Italy). The selected parameters were: a temperature of 100 °C for 60 min, with a microwave power of 600 W, a plant-to-solvent ratio of 1:40, and a solvent composition of 70% methanol. In contrast, for nitrates extraction, the selected parameters were: a temperature of 100 °C for 25 min, with a microwave power of 500 W, a plant-to-solvent ratio of 1:40, and a solvent composition of 100% ultrapure water. After all, for both extraction methods, the homogenates were centrifuged at 5000 rpm for 15 min (Centrifuge 5430 R, Eppendorf, Enfield, CT, USA), and the supernatants were collected and then filtered using 0.45 μm polytetrafluoroethylene (PTFE) microfilters (Corning, Steuben, NY, USA).

In total, 12 lettuce extracts were analyzed, as shown in Table 1: two lettuce cultivars (‘Carmesi’ and ‘Lugano’), three growth technologies (control—lettuce grown under natural oxygen levels—NOC, lettuce grown under elevated oxygen concentrations—EOC, and lettuce grown under elevated oxygen concentrations combined with LED light—EOC+LED), and two years of study, the winters of 2023 and 2024.

### 2.4. Characterization of Lettuce Bioactive Compounds

#### 2.4.1. Total Phenolic Content Assessment

The total phenolic content (TPC) was determined using the modified method established by Häuser et al. [27]. A volume of 50 µL of each hydroalcoholic lettuce extract was mixed with 50 µL of 1N Folin–Ciocalteu reagent and incubated in the dark at room temperature for 5 min. Then, the mixture was supplemented with 200 µL of 2% sodium carbonate. After 30 min of incubation, the absorbance was measured at λ750 nm using a POLARstar Omega microspectrophotometer (Biotron Healthcare, New Delhi, India). A 70% hydroalcoholic solution replaced the lettuce extract for the negative control, and the other reagents remained unchanged. Gallic acid (GA), in a linear range from 0.1 to 100 µg/mL, was used as a reference standard for TPC determination. The results were expressed as milligrams of gallic acid equivalents per kilogram of lettuce dry weight (mg GAE/kg DW). Lettuce extracts were analyzed in triplicate, and the results were presented as mean ± standard deviation.

#### 2.4.2. Total Flavonoid Content Assessment

The total flavonoid content (TFC) was determined using the modified aluminum chloride method established by Shi et al. [28]. Briefly, each lettuce extract was mixed with a 2% aluminum chloride solution at a 1:1 volume ratio. After 15 min of incubation in the dark at room temperature, the absorbance was measured at λ415 nm using a POLARstar Omega microspectrophotometer (Biotron Healthcare, New Delhi, India). A 70% hydroalcoholic solution replaced the lettuce extract for the negative control, and the other reagents remained unchanged. Quercetin (Q), in a linear range from 0.1 to 100 µg/mL, was used as a reference standard for TFC determination. The results were expressed as milligrams of quercetin equivalents per kilogram of lettuce dry weight (mg QE/kg DW). Lettuce extracts were analyzed in triplicate, and the results were presented as mean ± standard deviation.

#### 2.4.3. Polyphenolic Compounds by Ultra High-Performance Liquid Chromatography

Chromatographic analysis was performed on an Acquity Ultra High-Performance Liquid Chromatography (UHPLC) I Class system coupled to a Photodiode Array detector (PDA) (Waters Corporation, Milford, MA, USA) for each lettuce extract. Chromatographic separation was performed on a Zorbax Eclipse Plus C18 column (4.6 mm × 150 mm, with a particle size of 5 µm). The mobile phase consisted of (A) 0.1% formic acid in water and (B) 0.1% formic acid in acetonitrile, and the flow rate was 0.8 mL/min. The column temperature was maintained at 30 °C and the autosampler at 25 °C, using an injection volume of 10 μL. Elution conditions were optimized: 0–100% (B) for 45 min. For the identification and quantification of polyphenolic compounds, seven reference compounds were used: chlorogenic acid, caffeic acid, syringic acid, p-coumaric acid, quercetin-3-O-glucopyranoside, kaempferol-3-O-rutinoside, and kaempferol-3-O-glucoside, in a linear range from 0 to 60 µg/mL. Chromatograms were recorded at λ280, λ310, λ325, and λ350 nm. Thus, λ280 nm was used for syringic acid, λ310 nm was used for p-coumaric acid, λ325 nm was used for chlorogenic acid and caffeic acid, and λ350 nm was used for quercetin-3-O-glucopyranoside, kaempferol-3-O-rutinoside, and kaempferol-3-O-glucoside. The results were expressed as milligrams per kilogram of lettuce dry weight (mg/kg DW). Lettuce extracts were analyzed in triplicate, and the results were presented as mean ± standard deviation.

### 2.5. Characterization of Lettuce Antioxidant Activities

#### 2.5.1. Free Radical-Scavenging Activity Using 2,2-Diphenyl-1-picrylhydrazyl

The 2,2-diphenyl-1-picrylhydrazyl (DPPH) assay is based on the ability of antioxidants to scavenge the DPPH radical through a hydrogen atom transfer mechanism. A decrease in absorbance, proportional to the antioxidant activity, followed the reaction, changing the color from deep purple to pale yellow [29].

Briefly, various concentrations of each lettuce extract were mixed with a 200 µM DPPH methanolic solution at a 1:1 volume ratio. The mixtures were incubated in the dark at room temperature for 30 min. A 70% hydroalcoholic solution replaced the lettuce extract for the negative control, and the other reagents remained unchanged. Trolox (T), in a linear range from 0.1 to 100 µmol/mL, was used as a reference standard for DPPH scavenging activity. The absorbances were at λ517 nm using a POLARstar Omega microspectrophotometer (Biotron Healthcare, New Delhi, India). The antioxidant activity was expressed as micromoles of trolox equivalents per kilogram of lettuce dry weight (µmol TE/kg DW). Lettuce extracts were analyzed in triplicate, and the results were presented as mean ± standard deviation.

Radical scavenging activity was expressed as percentage (RSA%) and determined with the following equation:

(RSA%) = (Abs _negative control_ − Abs _lettuce extract_)/Abs _negative control_ × 100 (1)

The 50% inhibitory concentration, also known as IC_50_, was determined using the RSA% from various concentrations of lettuce extract [30].

#### 2.5.2. Ferric Reduction Antioxidant Power

The Ferric Reduction Antioxidant Power (FRAP) assay is a method for determining the antioxidant capacity of antioxidants based on their redox potential. Ferric ions (Fe^3+^) are reduced to ferrous ions (Fe^2+^) by electron-donating antioxidants. The resulting Fe^2+^, mixed with 2,4,6-tris(2-pyridyl)-s-triazine (TPTZ), forms a blue-colored complex [31].

The FRAP assay was carried out according to Sutulienė et al. [32], with some modifications. The working solution of FRAP was prepared by mixing 100 mL of acetate buffer solution (250 mM, pH 3.6), 10 mL of TPTZ solution (10 mM in 40 mM HCl), and 10 mL of iron (III) chloride (20 mM). The working solution was mixed with each lettuce extract at a 1:4 volume ratio. The mixtures were read using a POLARstar Omega microspectrophotometer (Biotron Healthcare, New Delhi, India) at λ593 nm after being incubated in the dark at 37 °C for 30 min. A 70% hydroalcoholic solution replaced the lettuce extract for the negative control, and the other reagents remained unchanged. Trolox (T), in a linear range from 0.1 to 100 µmol/mL, was used as a reference standard for DPPH scavenging activity. The antioxidant activity was expressed as micromoles of trolox equivalents per kilogram of lettuce dry weight (µmol TE/kg DW). Lettuce extracts were analyzed in triplicate, and the results were presented as mean ± standard deviation.

#### 2.5.3. Cupric Ion Reducing Antioxidant Capacity

The Cupric Ion Reducing Antioxidant Capacity (CUPRAC) assay measures antioxidant activity by reducing a copper (II)—neocuproine complex to a copper (I)—neocuproine chelate [33]. The resulting complex exhibits a maximum absorbance, which increases in proportion to its antioxidant activity.

The CUPRAC assay was carried out according to [34], with some modifications. Every reagent was prepared as follows: 10 mM copper(II) chloride, 7 mM neocuproine, and a 1 M ammonium acetate buffer solution with a pH of 7. All reagents were prepared in ultrapure water, except for neocuproine, which was dissolved in methanol. Every lettuce extract was mixed with all four reagents at a 1:1:1:1 volume ratio. The mixtures were read using a POLARstar Omega microspectrophotometer (Biotron Healthcare, New Delhi, India) at λ450 nm after being incubated in the dark at room temperature for 30 min. A 70% hydroalcoholic solution replaced the lettuce extract for the negative control, and the other reagents remained unchanged. Trolox (T), in a linear range from 0.1 to 100 µmol/mL, was used as a reference standard for DPPH scavenging activity. The antioxidant activity was expressed as micromoles of trolox equivalents per kilogram of lettuce dry weight (µmol TE/kg DW). Lettuce extracts were analyzed in triplicate, and the results were presented as mean ± standard deviation.

#### 2.5.4. Antioxidant Index Assessment

The antioxidant index (AI) is a novel method of ranking different extracts based on their antioxidant activities [35]. Each lettuce extract received a score based on the mean relative percentage values derived from five experiments: DPPH, FRAP, CUPRAC, TPC, and TFC. To calculate the relative percentage, the highest result from each experiment was set as 100%, and the other results were then calculated proportionally. The resulting AI scores were then categorized into five classes: very low (0–19%), low (20–39%), medium (40–59%), high (60–79%), and very high (80–100%).

### 2.6. Nitrate Residue Assessment

The assessment of nitrate residues in each lettuce extract was determined in the presence of DMF reagent (2,6-dimethylphenol). To 100 µL of each lettuce extract, 50 µL of 2% DMF reagent in acetone, and 100 µL of a 9:1 volume ratio of sulfuric acid and phosphoric acid were added. After 30 min of incubation in the dark at room temperature, the absorbance was measured at λ345 nm using a POLARstar Omega microspectrophotometer (Biotron Healthcare, New Delhi, India). Ultrapure water replaced the lettuce extract for the negative control, and the other reagents remained unchanged. Sodium nitrate (NaNO_3_), in a linear range from 0 to 20 µg/mL, was used as a reference standard. The results were expressed as milligrams of nitrate per kilogram of lettuce dry weight (mg NO_3_^−^/kg DW). Lettuce extracts were analyzed in triplicate, and the results were presented as mean ± standard deviation.

### 2.7. Statistical Analyses Assessment

All experiments were performed independently in triplicate (n = 3). The results were presented as average values with standard deviation (less than 10% of the average). The different lettuce growth technologies were analyzed by one-way ANOVA (*p* ≤ 0.05), using GraphPad Prism Software version 10.5.0 (Boston, MA, USA). Additionally, a Principal Component Analysis (PCA) was performed to examine the correlation between the lettuce extracts’ polyphenolic compounds and antioxidant activities using the RStudio tool, version: 2025.09.1+401.

## 3. Results

### 3.1. Lettuce Bioactive Compounds

#### 3.1.1. Total Phenolic Content

**Figure 1:**
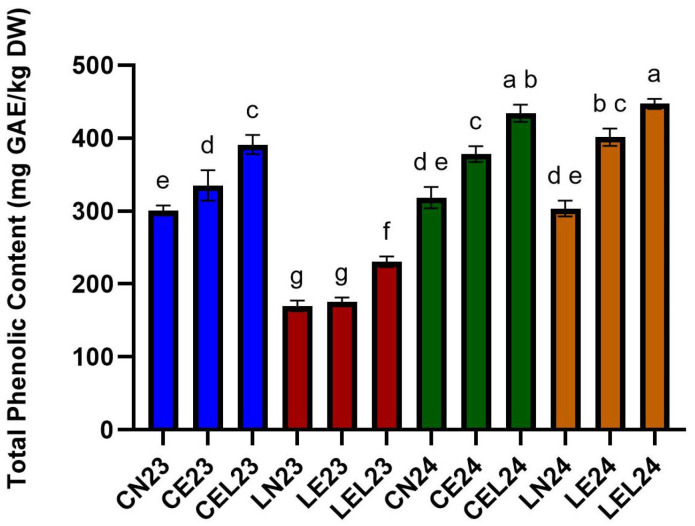
Total Phenolic Content of lettuce extracts expressed as milligrams of gallic acid equivalents per kilogram of lettuce dry weight (mg GAE/kg DW). Different letters indicate significant differences detected among lettuce extracts using One-way ANOVA (*p* < 0.05).

The total phenolic content (TPC), as shown in Figure 1, significantly increased when lettuce was grown under elevated oxygen concentrations (EOC) and elevated oxygen concentrations combined with LED light (EOC+LED), compared to the control, lettuce grown under natural oxygen concentrations (NOC). For example, in 2023, for the Carmesi cultivar, the TPC increased by 30% when EOC+LED was used as the growth technology (CEL23—391.20 ± 12.99 mg GA/kg DW), and the TPC increased by 12% when EOC was used as the growth technology (335.21 ± 20.72 mg GA/kg DW), compared to NOC used as the growth technology (300.96 ± 6.74 mg GA/kg DW). Additionally, for the Lugano cultivar grown in 2023, the most notable increase in TPC, which showed a 36% increase, occurred with the EOC+LED growth technology (LEL 23—230.28 ± 7.36 mg GA/kg DW), followed by the EOC growth technology, which showed a 3% increase in TPC (175.63 ± 5.64 mg GA/kg DW), compared to NOC growth technology (169.94 ± 7.23 mg GA/kg DW).

In 2024, this trend continued, with the EOC+LED growth technology producing the most significant increase in TPC for both lettuce cultivars, Carmesi and Lugano. Compared to the control, the NOC growth technology (CN24—318.58 ± 14.48 mg GA/kg DW, and LN24—303.35 ± 10.84 mg GA/kg DW), the Carmesi cultivar showed a 36% increase in TPC (434.10 ± 11.74 mg GA/kg DW), and the Lugano cultivar showed a 48% increase in TPC (447.13 ± 6.97 mg GA/kg DW), when EOC+LED was used as grown technology, followed by the EOC growth technology that showed a 19% increase in TPC for the Carmesi cultivar (378.09 ± 10.80 mg GA/kg DW), and a 34% increase in TPC for the Lugano cultivar (401.34 ± 11.85 mg GA/kg DW). The highest TPC increase was observed for Lugano grown in 2024 under the EOC+LED growth technology (from LN24—303.35 ± 10.84 mg GA/kg DW to LEL24—447.13 ± 6.97 mg GA/kg DW).

#### 3.1.2. Total Flavonoid Content

**Figure 2:**
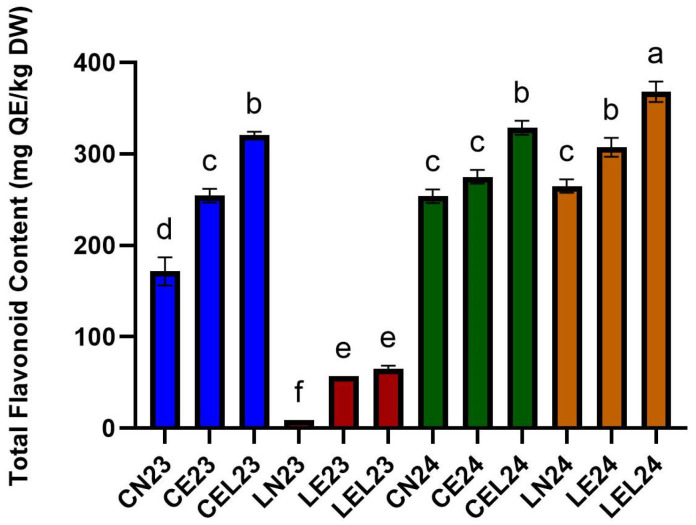
Total Flavonoid Content of lettuce extracts expressed as milligrams of quercetin equivalents per kilogram of lettuce dry weight (mg QE/kg DW). Different letters indicate significant differences detected among lettuce extracts using One-way ANOVA (*p* < 0.05).

As observed in the TPC, the total flavonoid content (TFC), as shown in Figure 2, increased significantly when lettuce was grown under EOC and EOC+LED growth technologies, compared to the control, the NOC growth technology. However, the Carmesi cultivar grown in 2023 exhibited the highest TFC when EOC+LED was used as the growth technology (CEL23—320.46 ± 3.83 mg Q/kg DW), representing an 87% increase compared to the NOC growth technology (CN23—171.60 ± 15.31 mg Q/kg DW). Additionally, the EOC growth technology represents a 48% increase in TFC (CE23—254.37 ± 7.39 mg Q/kg DW) compared to the NOC growth technology. In the case of the Lugano cultivar grown in 2023, the EOC+LED growth technology also presented the highest TFC (LEL23—64.88 ± 3.76 mg Q/kg DW), with a 631% increase compared to the NOC growth technology (LN23—8.88 ± 0.00 mg Q/kg DW). The Lugano cultivar grown under the EOC growth technology (56.90 ± 0.00 mg Q/kg DW) also exhibited a considerable increase in TPC, with a 541% increase compared to the NOC growth technology.

For both lettuce cultivars, Carmesi and Lugano, grown in 2024, the most effective growth technology was the EOC+LED, with a TFC increase of 30% for the Carmesi cultivar (CEL24—328.58 ± 7.66 mg Q/kg DW) and 39% for the Lugano cultivar (368.02 ± 11.28 mg Q/kg DW) compared to the NOC growth technology (CN24—253.68 ± 7.52 mg Q/kg DW, and LN24—264.82 ± 7.39 mg Q/kg DW). Additionally, the EOC growth technology represents an 8% increase in TFC for the Carmesi cultivar (CE24—274.95 ± 7.52 mg Q/kg DW) and a 16% increase in TFC for the Lugano cultivar (LE24—307.16 ± 10.48 mg Q/kg DW) compared to the NOC growth technology. The highest TFC increase was observed for Lugano grown in 2024 under the EOC+LED growth technology (from LN24—264.82 ± 7.39 mg Q/kg DW to LEL24—307.16 ± 10.48 mg Q/kg DW). Briefly, the EOC+LED growth technology showed a higher increase in the TPC and TFC of the two lettuce cultivars, Carmesi and Lugano, followed by the EOC growth technology and the NOC growth technology.

#### 3.1.3. Polyphenolic Compounds by UPLC

**Figure 3:**
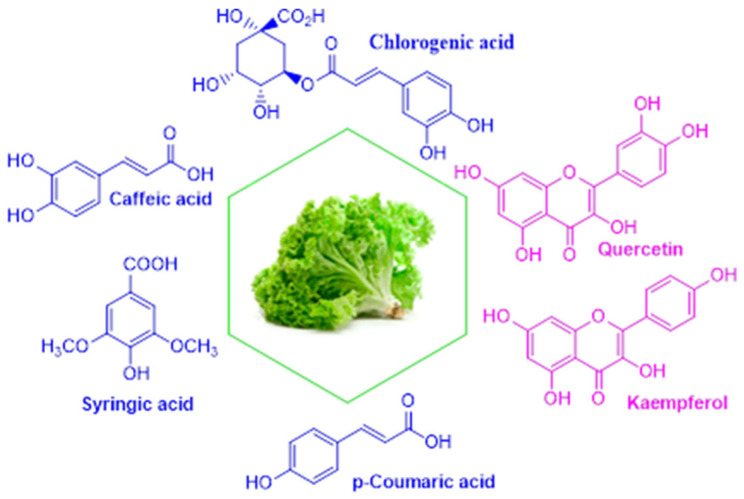
Polyphenolic compounds present in lettuce extracts.

A total of seven polyphenolic compounds were identified and quantified in lettuce extracts, including four phenolic acids (chlorogenic acid, caffeic acid, syringic acid, and p-coumaric acid) and three glycosylated flavonoids (quercetin-3-O-glucopyranoside, kaempferol-3-O-rutinoside, and kaempferol-3-O-glucoside), as shown in Figure 3. To better understand the accumulation of phenolic acids and flavonoids in lettuce in relation to adapted growth technologies, the differences between EOC and EOC+LED growth technologies, compared to the control NOC growth technology, were examined using modern chromatographic analysis, as shown in Table 2.

For both lettuce cultivars, Carmesi and Lugano, all polyphenolic compounds exhibit higher concentrations in the EOC+LED growth technology, followed by the EOC growth technology, and the lowest concentration in the NOC growth technology. The exception is caffeic acid, where different adapted growth technologies do not have a positive impact, and its concentration remains the same in all growth technologies, for both lettuce cultivars. The most abundant polyphenolic compounds are chlorogenic acid and quercetin-3-O-glucopyranoside in both lettuce cultivars when lettuce was grown under the EOC+LED growth technology: CEL24—926.06 ± 16.32 mg/kg, and LEL24—842.46 ± 14.37 mg/kg for chlorogenic acid, and CEL24—760.38 ± 0.44 mg/kg, and LEL24—853.48 ± 16.20 mg/kg for quercetin-3-O-glucopyranoside. Additionally, for the Lugano cultivar, the EOC growth technology has a positive impact on polyphenolic compounds, resulting in the highest concentrations of chlorogenic acid (LE24—742.14 ± 16.35 mg/kg) and quercetin-3-O-glucopyranoside (LE24—775.96 ± 0.00 mg/kg). These aspects are also the same for the other two glycosylated flavonoids, kaempferol-3-O-rutinoside (LE24—315.02 ± 0.85 mg/kg) and kaempferol-3-O-glucoside (LE24—509.20 ± 9.73 mg/kg). After all, the highest concentration of polyphenolic compounds was found in Lugano, grown in 2024 under the EOC+LED growth technology.

### 3.2. Lettuce Antioxidant Activities

#### 3.2.1. Antioxidant Equivalents

Regarding the antioxidant activity, expressed as antioxidant equivalents, of the lettuce extracts, three antioxidant assays were conducted, namely DPPH, FRAP, and CUPRAC, as shown in Table 3. The DPPH assay reported that the best lettuce extract with higher antioxidant equivalents was the Lugano grown in 2024 under EOC+LED growth technology (LEL24—3696.09 ± 21.66 µmol TE/kg DW), followed by Carmesi grown in 2023 under EOC+LED growth technology (CEL24—3485.40 ± 20.23 µmol TE/Kg DW). Consequently, the lettuce extract that has the highest antioxidant equivalents, as determined by the FRAP assay, was Carmesi grown in 2024 under EOC+LED growth technology (CEL24—1581.87 ± 20.75 µmol TE/kg DW), followed by the Carmesi grown in 2023 under EOC+LED growth technology (CEL23—1442.49 ± 35.27 µmol TE/kg DW). Lastly, the highest antioxidant equivalents, as determined by the CUPRAC assay, were also those from lettuce grown in 2024 under the EOC+LED growth technology, from both lettuce cultivars, Carmesi (CEL24—3313.76 ± 80.17 µmol TE/kg), and Lugano (LEL24—3255.63 ± 12.76 µmol TE/kg). The overall results of the antioxidant assays indicated that both lettuce cultivars showed significant improvement in bioactive compound accumulation and their antioxidant activities under the EOC+LED growth technology.

#### 3.2.2. Antioxidant Index

The antioxidant index (AI) was calculated to integrate the results of all antioxidant assays, identifying the lettuce extract with the highest overall antioxidant activities. Moreover, it is an easy way to classify lettuce extracts into five categories: very low, low, moderate, high, and very high antioxidant activity. The highest AI, as shown in Table 4, was observed in Carmesi grown in 2024 under the EOC+LED growth technology (CEL24—from 89% to 100%), followed closely by Lugano grown under the same technology in 2024 (LEL24—from 73% to 100%). Once again, the adapted EOC+LED growth technology proved to be the best compared to the control, the NOC growth technology.

### 3.3. Nitrates Residue

**Figure 4:**
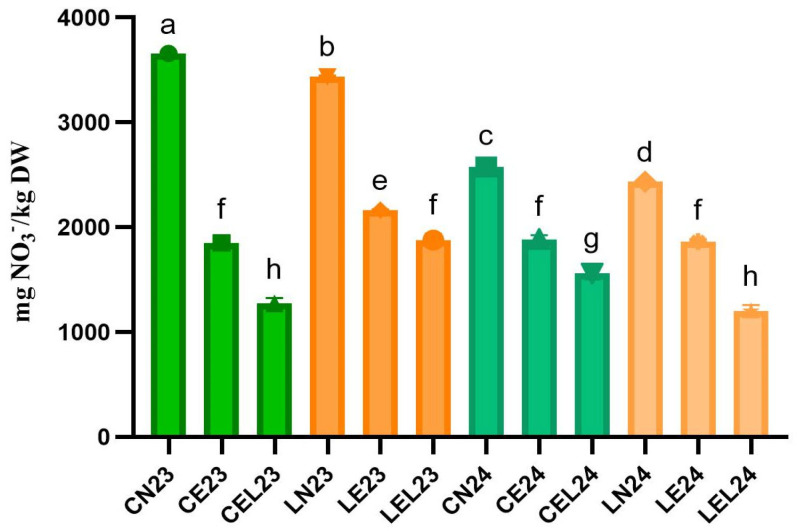
Nitrate residue in lettuce grown under different adapted growth technologies. Different letters indicate significant differences detected among lettuce extracts using One-way ANOVA (*p* < 0.05).

A comparative analysis of nitrate residue in lettuce grown under different growth technologies reveals significant differences in the lettuce’s ability to accumulate or metabolize nitrates, with values ranging from 1199.23 ± 58.19 to 1562.48 ± 60.35 mg nitrate/kg DW for EOC+LED growth technology, from 1851.22 ± 46.50 to 2163.93 ± 9.19 mg nitrate/kg DW for EOC growth technology, and compared to the control, NOC growth technology, with values ranging from 2436.58 ± 6.07 to 3658.93 ± 1.38 mg nitrate/kg DW (Figure 4). Comparing the values between 2023 and 2024, a clear reduction in nitrate content is observed in lettuce grown under EOC and EOC+LED growth technologies, compared to the control, NOC growth technology.

### 3.4. Statistical Analyses

**Figure 5:**
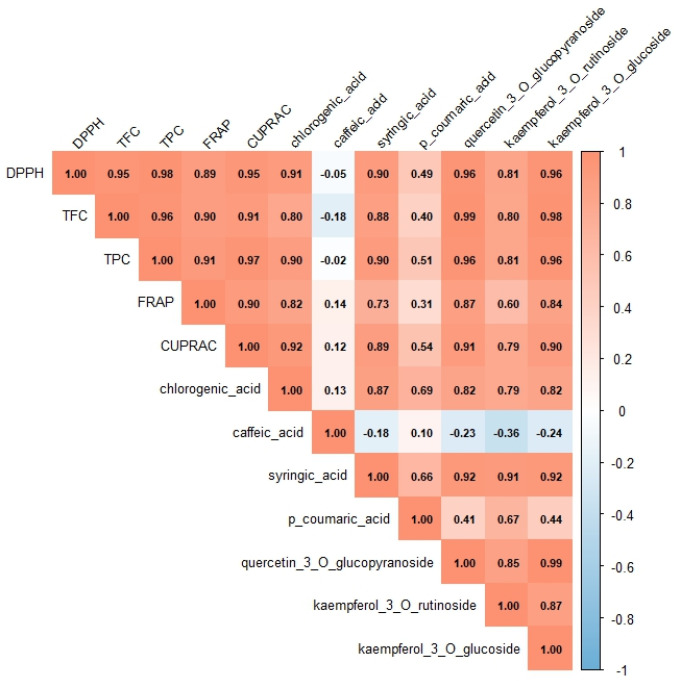
Pearson correlation matrix of lettuce extracts’ polyphenolic compounds and their antioxidant activities.

A Pearson correlation assay was performed to investigate the potential relationship between bioactive compound content and antioxidant activities in lettuce extracts (Figure 5). Results showed that TPC, TFC, and individual polyphenolic compounds were perfectly correlated with antioxidant activities, as measured by DPPH, FRAP, and CUPRAC assays. Furthermore, the findings indicated that polyphenolic compounds such as chlorogenic acid, syringic acid, quercetin-3-O-glucopyranoside, and kaempferol-3-O-glucoside are perfectly correlated with DPPH antioxidant activity. A perfect correlation was also observed between chlorogenic acid, quercetin-3-O-glucopyranoside, and kaempferol-3-O-glucoside and their antioxidant activity as measured by the FRAP assay. Lastly, chlorogenic acid, syringic acid, quercetin-3-O-glucopyranoside, and kaempferol-3-O-glucoside demonstrated a perfect correlation with CUPRAC antioxidant activity. A strong correlation was observed between syringic acid and kaempferol-3-O-rutinoside, as well as their antioxidant activity, as measured by the FRAP assay. Otherwise, p-coumaric acid demonstrates a moderate correlation link to DPPH and CUPRAC antioxidant activities. These correlations indicate that polyphenolic compounds play a major role in determining the antioxidant capacity of lettuce extracts, particularly under adapted growth technologies. The close association between glycosylated flavonoids and antioxidant assays suggests an enhanced secondary metabolic response linked to oxygen enrichment and supplemental LED lighting.

**Figure 6:**
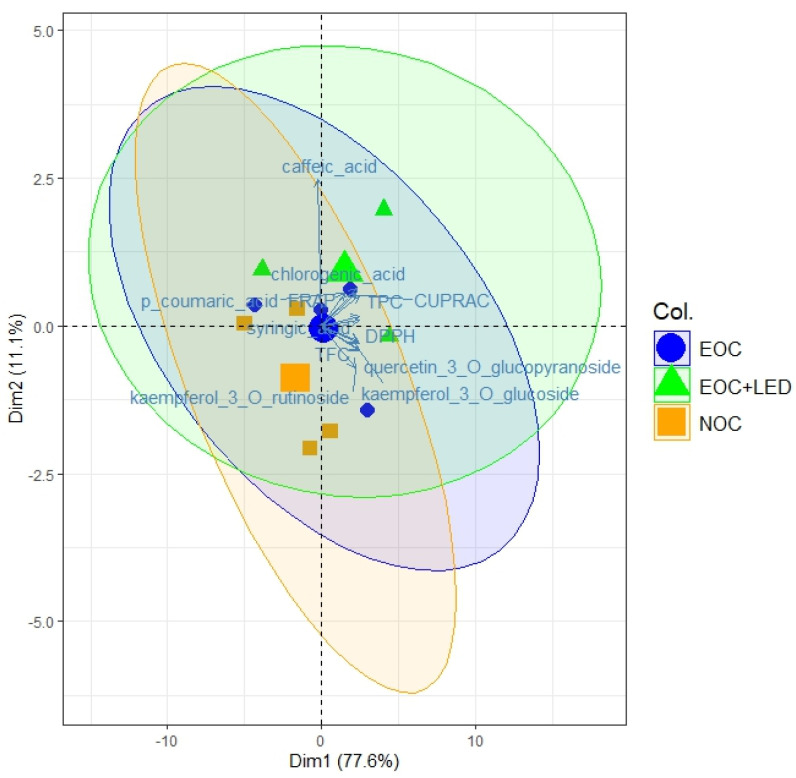
Principal component analysis (PCA) plot of lettuce extracts’ polyphenolic compounds and their antioxidant activities.

Principal component analysis (PCA) was used to explore correlations between different lettuce-adapted growth technologies, EOC and EOC+LED, compared to the control, the NOC growth technology (Figure 6). In the PCA biplot, the first principal component (PC1) accounted for 77.6% of the total variance, whereas the second component (PC2) reflected 11.1% of the total variance. Positive correlations are indicated by larger circles with a green tint for the EOC+LED growth technology and a purple tint for EOC growth technology, while the smaller circle with an orange tint shows negative correlations for the NOC growth technology. Briefly, EOC+LED growth technology significantly modified the content of bioactive compounds and their antioxidant activities. Additionally, EOC+LED growth technology indicates a strong stimulation of glycosylated flavonoids accumulation, such as quercetin-3-O-glucopyranoside, kaempferol-3-O-rutinoside, and kaempferol-3-O-glucoside. Otherwise, the EOC growth technology did not cause a significant change in the content of bioactive compounds and their antioxidant activities compared to the NOC growth technology. PCA further supported these findings by clearly separating lettuce cultivars according to cultivation technology. The EOC+LED growth technology was primarily associated with higher glycosylated flavonoid loadings and antioxidant activity, indicating strong stimulation of secondary metabolite biosynthesis. In contrast, the control growth technology (NOC) clustered separately, reflecting lower levels of bioactive compounds and antioxidant capacity. This multivariate pattern highlights the combined effect of elevated oxygen availability and LED lighting on lettuce metabolic regulation.

## 4. Discussion

Light is one of the most crucial factors necessary for lettuce photosynthesis and is a significant aspect in controlled-environment agriculture, particularly for hydroponic systems where artificial lamps are the sole light source for lettuce growth [36,37]. The elevated oxygen concentration, combined with LED red-blue light, emerged as the most effective technology for promoting overall lettuce growth, with notable accumulation of bioactive compounds. These results align with recent studies that emphasize the synergistic effects of LED red-blue light on enhancing photosynthetic activity and the accumulation of bioactive compounds in leafy vegetables [38].

The enhanced accumulation of bioactive compounds observed under elevated oxygen availability, combined with red–blue LED lighting, can be explained by synergistic effects on lettuce physiological and metabolic processes. Increased dissolved oxygen in the root zone improves root respiration and energy metabolism, supporting more efficient nutrient uptake and reducing hypoxic stress commonly encountered in hydroponic systems. Improved root oxygenation has been shown to positively influence plant metabolic balance and secondary metabolite accumulation in leafy vegetables [39,40]. At the same time, red–blue LED lighting directly modulates photosynthetic efficiency and activates light-responsive metabolic pathways involved in secondary metabolism. Blue light has been reported to stimulate phenylpropanoid metabolism and phenolic compound biosynthesis, while red light enhances photosynthetic carbon fixation, increasing the availability of carbon skeletons required for secondary metabolite production. These light-induced physiological responses have been associated with increased polyphenol and flavonoid accumulation and enhanced antioxidant activity in lettuce [41,42].

The increases in total phenolic and flavonoid contents observed in the present study under elevated oxygen availability and combined red–blue LED lighting are consistent with previous reports highlighting the role of controlled environmental conditions in enhancing lettuce phytochemical profiles. Several studies have demonstrated that red–blue LED lighting promotes the accumulation of phenolic compounds and antioxidant activity in lettuce by modulating photosynthetic efficiency and secondary metabolism [43,44].

In addition to light regulation, recent literature emphasizes the importance of oxygen availability in hydroponic systems. Improved root-zone oxygenation has been associated with enhanced nutrient uptake, delayed senescence, and improved nutritional quality in leafy vegetables [45]. Our findings align with these observations, showing that elevated oxygen conditions, particularly when combined with LED lighting, can further stimulate the accumulation of antioxidant-related metabolites.

Total phenolic compounds, including phenolic acids and flavonoids, play a key role as bioactive compounds in lettuce [46]. Our results suggest that the TPC and TFC increased considerably when the EOC and EOC+LED growth technologies were used, compared to the control growth technology, NOC. The antioxidant activities, as measured by DPPH, FRAP, and CUPRAC, in lettuce cultivars Carmesi and Lugano, have been attributed to their higher polyphenolic compounds, such as chlorogenic acid, caffeic acid, syringic acid, and p-coumaric acid, quercetin-3-O-glucopyranoside, kaempferol-3-O-rutinoside, and kaempferol-3-O-glucoside, which were improved with the use of EOC and EOC+LED growth technologies were used.

Lettuce nitrate residue is a primary concern due to its potential to convert into nitrites and nitrosamines, which can have a negative impact on human health [47]. Nitrate residue in lettuce is subjected to dynamic changes depending on genetic and environmental factors. Nitrate amounts tend to rise in lettuce leaf tissues when the rate of uptake exceeds the chemical reduction [48,49]. In general, lettuce from greenhouses tended to accumulate higher amounts of nitrates, due to the use of nitrogen-based fertilizers and controlled environmental conditions such as temperature and light. In this study, the EOC and EOC+LED growth technologies showed a significant reduction in nitrate residue compared to the control, the NOC growth technology. In brief, lettuce-adapted growth technologies are effective in reducing nitrate residue in lettuce. These aspects suggest compliance with food safety standards. Therefore, these results underscore the importance of rigorous monitoring of agricultural inputs and adherence to the maximum permissible limit for nitrates in lettuce. In previous research studies, this observation was also concluded, showing that the LED red-blue growth technology reduces nitrate residue in lettuce [7,50].

Principal component analysis (PCA) and Pearson correlation matrix further clarify the relationships between the accumulation of bioactive compounds, their antioxidant activities, and lettuce-adapted growth technologies. Additionally, the correlation plot also revealed a strong positive correlation between the accumulation of glycosylated flavonoids and lettuce growth under adapted growth technologies.

Although this study provides useful insights into the effects of elevated oxygen availability and red–blue LED lighting on lettuce quality, some limitations should be acknowledged. The research was performed on two lettuce cultivars under specific greenhouse conditions, which may limit the extrapolation of the results to other cultivars or growing environments. Future studies should evaluate a broader range of lettuce cultivars and cultivation conditions to further confirm the applicability of these findings.

## 5. Conclusions

Elevated oxygen concentrations (EOC) and elevated oxygen concentrations combined with LED red-blue light (EOC+LED) can improve the accumulation of lettuce bioactive compounds and increase their antioxidant activities in two lettuce cultivars, Carmesi and Lugano. Nevertheless, the adapted growth technologies, EOC and EOC+LED, resulted in a lower nitrate content than the European limits for lettuce grown in greenhouses during the winter period. Elevated oxygen concentrations combined with LED red-blue light for lettuce growth is a growing field for both research and commercial applications.

## Figures and Tables

**Table 1:** Codification of lettuce extracts.

Extract Code	Cultivar	Growth Technology	Year
**CN23**	Carmesi	NOC	2023
**CE23**	Carmesi	EOC	2023
**CEL23**	Carmesi	EOC+LED	2023
**LN23**	Lugano	NOC	2023
**LE23**	Lugano	EOC	2023
**LEL23**	Lugano	EOC+LED	2023
**CN24**	Carmesi	NOC	2024
**CE24**	Carmesi	EOC	2024
**CEL24**	Carmesi	EOC+LED	2024
**LN24**	Lugano	NOC	2024
**LE24**	Lugano	EOC	2024
**LEL24**	Lugano	EOC+LED	2024

NOC—natural oxygen levels, EOC—elevated oxygen concentrations, and EOC+LED—elevated oxygen concentrations combined with LED light.

**Table 2:** Polyphenolic compounds identified in lettuce extracts by UHPLC. Different letters indicate significant differences detected among extracts using One-way ANOVA (*p* < 0.05).

Extract Code	Polyphenolic Compounds						
	Chlorogenic Acid	Caffeic Acid	Syringic Acid	p-Coumaric Acid	Quercetin-3-O-glucopyranoside	Kaempferol-3-O-rutinoside	Kaempferol-3-O-glucoside
	λ (nm)						
	325	325	280	310	350	350	350
	Retention Time (min)						
	16.664	17.336	17.466	21.184	23.410	23.656	24.300
**CN23**	243.58 ± 3.10 ^g^	44.08 ± 0.34	70.68 ± 2.38 ^d^	41.80 ± 0.18 ^gh^	422.18 ± 13.07 ^f^	69.54 ± 0.00 ^h^	218.50 ± 0.33 ^h^
**CE23**	373.92 ± 5.29 ^e^	44.08 ± 1.77	79.04 ± 0.70 ^c^	42.94 ± 0.05 ^ef^	568.48 ± 10.32 ^e^	91.96 ± 1.11 ^g^	338.96 ± 0.00 ^g^
**CEL23**	490.20 ± 4.16 ^d^	44.46 ± 2.61	83.60 ± 0.71 ^c^	43.70 ± 0.11 ^e^	696.54 ± 5.11 ^c^	101.08 ± 0.02 ^f^	430.16 ± 3.78 ^d^
**LN23**	61.56 ± 0.90 ^h^	44.08 ± 0.40	54.34 ± 3.98 ^f^	41.04 ± 0.05 ^h^	99.94 ± 3.24 ^h^	45.22 ± 0.24 ^i^	59.66 ± 5.33 ^j^
**LE23**	66.50 ± 0.50 ^h^	44.08 ± 0.72	61.18 ± 1.17 ^e^	47.50 ± 0.65 ^c^	110.20 ± 0.05 ^h^	45.98 ± 0.77 ^i^	59.66 ± 0.00 ^j^
**LEL23**	69.54 ± 0.30 ^h^	44.46 ± 0.01	62.70 ± 0.97 ^e^	48.26 ± 0.48 ^c^	132.24 ± 3.11 ^g^	46.36 ± 0.00 ^i^	72.20 ± 1.99 ^i^
**CN24**	302.86 ± 10.20 ^f^	42.94 ± 0.79	99.56 ± 0.69 ^b^	46.36 ± 0.11 ^d^	661.20 ± 5.39 ^d^	228.76 ± 1.77 ^d^	376.20 ± 0.00 ^e^
**CE24**	500.84 ± 2.68 ^d^	44.46 ± 0.02	101.84 ± 1.19 ^b^	47.88 ± 0.03 ^c^	682.10 ± 0.00 ^cd^	231.42 ± 0.54 ^d^	451.44 ± 0.81 ^c^
**CEL24**	926.06 ± 16.32 ^a^	44.84 ± 0.05	101.84 ± 3.08 ^b^	52.06 ± 0.00 ^a^	760.38 ± 0.44 ^b^	295.64 ± 0.88 ^c^	461.32 ± 1.66 ^c^
**LN24**	30.20 ± 21.69 ^f^	42.94 ± 0.86	73.34 ± 1.50 ^d^	42.56 ± 0.00 ^fg^	567.72 ± 5.89 ^e^	204.82 ± 6.32 ^e^	363.66 ± 2.33 ^f^
**LE24**	742.14 ± 16.35 ^c^	42.94 ± 1.72	101.46 ± 8.32 ^b^	50.16 ± 0.07 ^b^	775.96 ± 0.00 ^b^	315.02 ± 0.85 ^b^	509.20 ± 9.73 ^b^
**LEL24**	842.46 ± 14.37 ^b^	43.70 ± 0.03	114.76 ± 0.00 ^a^	50.54 ± 0.65 ^b^	853.48 ± 16.20 ^a^	321.86 ± 0.12 ^a^	544.16 ± 4.99 ^a^

**Table 3:** Antioxidant equivalents of lettuce extracts. Different letters indicate significant differences detected among extracts using One-way ANOVA (*p* < 0.05).

Extract Code	DPPH IC_50_	DPPH (µmolTE/kg DW)	FRAP (µmolTE/kg DW)	CUPRAC (µmolTE/kg DW)	TPC (mgGA/kg DW)	TFC (mgQ/kg DW)
**CN23**	7.67 ± 0.14	1772.31 ± 5.23 ^h^	802.82 ± 31.12 ^f^	1732.90 ± 39.43 ^e^	300.96 ± 6.74 ^e^	171.60 ± 15.31 ^d^
**CE23**	5.12 ± 0.11	2645.48 ± 19.52 ^f^	1062.13 ± 1.52 ^d^	1742.42 ± 11.57 ^e^	335.21 ± 20.72 ^d^	254.37 ± 7.39 ^c^
**CEL23**	5.19 ± 0.12	2781.05 ± 25.28 ^e^	1442.49 ± 35.27 ^b^	2237.55 ± 51.26 ^c^	391.20 ± 12.99 ^c^	320.46 ± 3.83 ^b^
**LN23**	41.01 ± 1.40	199.64 ± 15.43 ^k^	194.12 ± 7.75 ^h^	397.75 ± 0.96 ^h^	169.94 ± 7.23 ^g^	8.88 ± 0.00 ^f^
**LE23**	29.38 ± 0.61	396.29 ± 10.83 ^j^	208.36 ± 18.59 ^h^	503.51 ± 18.65 ^h^	175.63 ± 5.64 ^g^	56.90 ± 0.00 ^e^
**LEL23**	26.94 ± 0.40	513.33 ± 29.28 ^i^	220.78 ± 4.57 ^h^	670.75 ± 0.00 ^g^	230.28 ± 7.36 ^f^	64.88 ± 3.76 ^e^
**CN24**	7.240 ± 0.32	1904.83 ± 20.57 ^g^	805.38 ± 26.34 ^f^	1647.11 ± 18.65 ^e^	318.58 ± 14.48 ^de^	253.68 ± 7.52 ^c^
**CE24**	4.64 ± 0.02	3022.35 ± 10.83 ^d^	847.01 ± 17.04 ^f^	2434.02 ± 40.24 ^b^	378.09 ± 10.80 ^c^	274.95 ± 7.52 ^c^
**CEL24**	3.88 ± 0.08	3485.40 ± 20.23 ^b^	1581.87 ± 20.75 ^a^	3313.76 ± 80.17 ^a^	434.10 ± 11.74 ^ab^	328.58 ± 7.66 ^b^
**LN24**	7.70 ± 0.07	1785.12 ± 10.10 ^h^	708.16 ± 9.13 ^g^	1396.20 ± 46.26 ^f^	303.35 ± 10.84 ^de^	264.82 ± 7.39 ^c^
**LE24**	3.89 ± 0.11	3428.53 ± 15.08 ^c^	977.75 ± 30.21 ^e^	2101.65 ± 53.77 ^d^	401.34 ± 11.85 ^bc^	307.16 ± 10.48 ^b^
**LEL24**	3.75 ± 0.04	3696.09 ± 21.66 ^a^	1155.93 ± 4.65 ^c^	3255.63 ± 12.76 ^a^	447.13 ± 6.97 ^a^	368.02 ± 11.28 ^a^

**Table 4:** Comparative analysis of lettuce extracts based on antioxidant index ratings.

Extract Code	Rel% DPPH	Rel% FRAP	Rel% CUPRAC	Rel% TPC	Rel% TFC	AI	Category
**CN23**	48	51	52	67	47	53	Medium
**CE23**	72	67	53	75	69	67	High
**CEL23**	75	91	68	87	87	82	Very high
**LN23**	5	12	12	38	2	14	Very low
**LE23**	11	13	15	39	15	22	Low
**LEL23**	14	14	20	52	18	27	Low
**CN24**	52	51	50	71	69	59	Medium
**CE24**	82	54	73	85	75	74	High
**CEL24**	94	100	100	97	89	96	Very high
**LN24**	48	45	42	68	72	55	Medium
**LE24**	93	62	63	90	83	78	High
**LEL24**	100	73	98	100	100	94	Very high

Rel%—relative percentage.

## Data Availability

The original contributions presented in the study are included in the article. Further inquiries can be directed to the corresponding author.

## References

[1] Hernández-Adasme C., Silva H., Escalona V. (2021). In-door germination and seedling growth of green and red lettuce under LED-light spectrum and subsequent effect on baby leaf lettuce. Ital. J. Agron..

[2] Sumi M.J., Jahan N., Thamid S.S., Tarik M.E.I., Hassannejad S., Rahimi M., Imran S. (2025). LED light effect on growth, pigments, and antioxidants of lettuce (*Lactuca sativa* L.) baby greens. BMC Plant Biol..

[3] Shi M., Gu J., Wu H., Rauf A., Emran T.B., Khan Z., Mitra S., Aljohani A.S.M., Alhumaydhi F.A., Al-Awthan Y.S. (2022). Phytochemicals, Nutrition, Metabolism, Bioavailability, and Health Benefits in Lettuce—A Comprehensive Review. Antioxidants.

[4] Food and Agriculture Organization of the United Nations. https://www.fao.org/faostat/en/#data/QCL.

[5] Grzegorzewska M., Badełek E., Matysiak B., Kaniszewski S., Dyśko J., Kowalczyk W., Wrzodak A., Szwejda-Grzybowska J. (2023). Assessment of romaine lettuce cultivars grown in a vertical hydroponic system at two levels of LED light intensity. Sci. Hortic..

[6] Stoica C.M., Velcea M., Chira L., Jerca O.I., Velea M.A., Drăghici E.M. (2022). The Nutrient Solution Oxygenation Influence on the Growth of the Species *Lactuca sativa* L. Root System Cultivated in the Nutrient Film Technique (NFT) System. Horticulture.

[7] Shen W., Zhang W., Li J., Huang Z., Tao Y., Hong J., Zhang L., Zhou Y. (2024). Pre-harvest short-term continuous LED lighting improves the nutritional quality and flavor of hydroponic purple-leaf lettuce. Sci. Hortic..

[8] Łaźny R., Mirgos M., Przybył J.L., Wójcik-Gront E., Bella S., Gajc-Wolska J., Kowalczyk W., Nowak J.S., Kunka M., Kowalczyk K. (2024). Effect of lignite substrate compared to mineral wool and supplementary lighting with HPS and LED on growth, plant photosynthetic activity, yield and fruit quality of greenhouse cucumber. Sci. Hortic..

[9] Liu J., Liu W. (2022). Regulation of accumulation and metabolism circadian rhythms of starch and sucrose in two leaf-color lettuces by red: Blue ratios of LED continuous light. Environ. Exp. Bot..

[10] Tsaballa A., Xanthopoulou A., Sperdouli I., Bantis F., Boutsika A., Chatzigeorgiou I., Tsaliki E., Koukounaras A., Ntinas G.K., Ganopoulos I. (2023). LED omics in Rocket Salad (*Diplotaxis tenuifolia*): Comparative Analysis in Different Light-Emitting Diode (LED) Spectrum and Energy Consumption. Plants.

[11] Fiore L., Cardarelli M., Lliuya J.C.L., Bonini P., Santelli P., Colla G. (2025). Nanobubble- and Microbubble Aeration Affect Leaf Quality Without Changing Yield of Lettuce Grown in Floating Systems. Horticulturae.

[12] Zhao Q., Dong J., Li S., Lei W., Liu A. (2024). Effects of micro/nano-ozone bubble nutrient solutions on growth promotion and rhizosphere microbial community diversity in soilless cultivated lettuces. Front. Plant Sci..

[13] Lan Y., Chu Q., Liu X., Xu S., Li D., Zhang C., He P., Feng X., Zhang H., Sha Z. (2025). Oxygen-nanobubble-loaded biochar increases soil carbon sequestration in rice paddies. Soil Environ. Health.

[14] Zheng K., Zeng H., Liu R., Wu L., Pan Y., Li J., Shang C. (2025). Research Progress on the Regulation of Plant Rhizosphere Oxygen Environment by Micro-Nano Bubbles and Their Application Prospects in Alleviating Hypoxic Stress. Agronomy.

[15] Wang W., Lin Z., Wang W., Shang M., Lv H., Zong Q., Li J., Liang B., Zhou W. (2023). Elicitation with hydrogen peroxide promotes growth, phenolic-enrichment, antioxidant activity and nutritional values of two hydroponic lettuce genotypes. Food Chem. X.

[16] Jin J., Wang T., Wang Y., Yao J., Song J. (2025). Synergistic Regulation of Light Intensity and Calcium Nutrition in PFAL-Grown Lettuce by Optimizing Morphogenesis and Nutrient Homeostasis. Phyton.

[17] Dai J., Mumper R.J. (2010). Plant Phenolics: Extraction, Analysis and Their Antioxidant and Anticancer Properties. Molecules.

[18] Llorach R., Martínez-Sánchez A., Tomás-Barberán F.A., Gil M.I., Ferreres F. (2008). Characterisation of polyphenols and antioxidant properties of five lettuce varieties and escarole. Food Chem..

[19] Boo H., Heo B., Gorinstein S., Chon S. (2011). Positive effects of temperature and growth conditions on enzymatic and antioxidant status in lettuce plants. Plant Sci..

[20] Bian Z.H., Yang Q.C., Liu W.K. (2015). Effects of light quality on the accumulation of phytochemicals in vegetables produced in controlled environments: A review. J. Sci. Food Agric..

[21] Song J., Huang H., Hao Y., Song S., Zhang Y., Su W., Liu H. (2020). Nutritional quality, mineral and antioxidant content in lettuce affected by interaction of light intensity and nutrient solution concentration. Sci. Rep..

[22] Zhang H., He H., Song W., Zheng L. (2023). Pre-Harvest UVB Irradiation Enhances the Phenolic and Flavonoid Content, and Antioxidant Activity of Green- and Red-Leaf Lettuce Cultivars. Horticulturae.

[23] Sakamoto M., Suzuki T. (2024). N-Acetylcysteine Mitigates Oxidative Stress Induced by Transplanting Lettuce Seedlings into a DFT Hydroponic System. Agronomy.

[24] Zhang L., Wang Z., Huang T., Peng J., Sun C., Qin Q., Song B., Contreras V.H.E., Li Y., Yang Q. (2025). Exogenous application of glycine/nitrate fertilizer at an appropriate ratio enhances lettuce nutritional quality. Sci. Hortic..

[25] Devlamynck R., Fernandes de Souza M., Bog M., Leenknegt J., Eeckhout M., Meers E. (2020). Effect of the growth medium composition on nitrate accumulation in the novel protein crop Lemna minor. Ecotoxicol. Environ. Saf..

[26] Wojciechowska R., Kołton A., Długosz-Grochowska O., Knop E. (2016). Nitrate content in *Valerianella* locusta L. Plants is affected by supplemental LED lighting. Sci. Hortic..

[27] Häuser H., Pilger A., Ulrichs C., Kätzel R. (2025). Phenolic Leaf Compounds in Ash Trees (*Fraxinus excelsior* L.) in the Context of Ash Dieback. Forests.

[28] Shi L., Zhao W., Yang Z., Subbiah V., Rasul Suleria H.A. (2022). Extraction and characterization of phenolic compounds and their potential antioxidant activities. Environ. Sci. Pollut. Res..

[29] Munteanu I.G., Apetrei C. (2021). Analytical Methods Used in Determining Antioxidant Activity: A Review. Int. J. Mol. Sci..

[30] Chen Z., Bertin R., Froldi G. (2013). EC50 estimation of antioxidant activity in DPPH assay using several statistical programs. Food Chem..

[31] Benzie F.F., Devaki M., Apak R., Capanoglu E., Shahidi F. (2017). The ferric reducing/antioxidant power (FRAP) assay for non-enzymatic antioxidant capacity: Concepts, procedures, limitations and applications. Measurement of Antioxidant Activity & Capacity.

[32] Sutulienė R., Laužikė K., Pukas T., Samuolienė G. (2022). Effect of Light Intensity on the Growth and Antioxidant Activity of Sweet Basil and Lettuce. Plants.

[33] Apak R., Güçlü K., Özyürek M., Karademir S.E. (2004). Novel total antioxidant capacity index for dietary polyphenols and vitamins C and E, using their cupric ion reducing capability in the presence of neocuproine (CUPRAC method). J. Agric. Food Chem..

[34] Akar Z., Burnaz N.A. (2019). A new colorimetric method for CUPRAC assay with using of TLC plate. LWT.

[35] Imtiaz F., Ahmed D., Mohammed O.A., Younas U., Iqbal M. (2024). Optimized recovery of phenolic and flavonoid compounds from medicinal plant extracts for enhanced antioxidant activity: A mixture design approach. Results Chem..

[36] Mohamed S.J., Rihan H.Z., Aljafer N., Fuller M.P. (2021). The Impact of Light Spectrum and Intensity on the Growth, Physiology, and Antioxidant Activity of Lettuce (*Lactuca sativa* L.). Plants.

[37] Chen X., Wang L., Li T., Yang Q., Guo W. (2019). Sugar accumulation and growth of lettuce exposed to different lighting modes of red and blue LED light. Sci. Rep..

[38] Bergstrand K.J., Harju V., Tossavainen M. (2025). End-of-production light treatments as a tool for controlling chemical composition of herbs and lettuce. Sci. Hortic..

[39] Moreno Roblero M.J., Pineda J., Colinas León M.T., Sahagún Castellanos J. (2020). Oxygen in the root zone and its effect on plants. Rev. Mex. Cienc. Agríc.

[40] Baiyin B., Tagawa K., Yamada M., Wang X., Yamada S., Yamamoto S., Ibaraki Y. (2021). Study on Plant Growth and Nutrient Uptake under Different Aeration Intensity in Hydroponics with the Application of Particle Image Velocimetry. Agriculture.

[41] Li Q., Kubota C. (2009). Effects of supplemental light quality on growth and phytochemicals of baby leaf lettuce. Environ. Exp. Bot..

[42] Johkan M., Shoji K., Goto F., Hashida S., Yoshihara T. (2012). Blue light-emitting diode light irradiation of seedlings improves seedling quality and growth after transplanting in red leaf lettuce. HortScience.

[43] Samuolienė G., Sirtautas R., Brazaitytė A., Duchovskis P. (2012). LED lighting and seasonality effects antioxidant properties of baby leaf lettuce. Food Chem..

[44] Lin K.H., Huang M.Y., Huang W.D., Hsu M.H., Yang Z.W., Yang C.M. (2013). The effects of red, blue, and white light-emitting diodes on the growth, development, and edible quality of hydroponically grown lettuce (*Lactuca sativa* L. *var. capitata*). Sci. Hortic..

[45] Leibar-Porcel E., McAinsh M.R., Dodd I.C. (2020). Elevated Root-Zone Dissolved Inorganic Carbon Alters Plant Nutrition of Lettuce and Pepper Grown Hydroponically and Aeroponically. Agronomy.

[46] Hameed M.K., Umar W., Razzaq A., Wei S., Niu Q., Huang D., Chang L. (2023). Quantification of total polyphenols, antioxidants, anthocyanins and secondary metabolites by UPLC VION IMS QTOF MS/MS analysis in green and red lettuce cultivars. Sci. Hortic..

[47] Kyriacou M.C., Soteriou G.A., Colla G., Rouphael Y. (2019). The occurrence of nitrate and nitrite in Mediterranean fresh salad vegetables and its modulation by preharvest practices and postharvest conditions. Food Chem..

[48] Chen B., Wang Z., Li S., Wang G., Song H., Wang X. (2004). Effects of nitrate supply on plant growth, nitrate accumulation, metabolic nitrate concentration and nitrate reductase activity in three leafy vegetables. Plant Sci..

[49] Zayed O., Hewedy O.A., Abdelmoteleb A., Ali M., Youssef M.S., Roumia A.F., Seymour D., Yuan Z.-C. (2023). Nitrogen Journey in Plants: From Uptake to Metabolism, Stress Response, and Microbe Interaction. Biomolecules.

[50] Viršilė A., Brazaitytė A., Jankauskienė J., Miliauskienė J., Vaštakaitė V., Odminytė I., Samuolienė G. (2018). Pre-harvest LED lighting strategies for reduced nitrate contents in leafy vegetables. Zemdirbyste-Agriculture.

